# The Influence of Substrate Topography on the Plasma Jet Flow in the Substrate Boundary Layer under Suspension Plasma Spray Conditions: A Numerical Approach

**DOI:** 10.1007/s11666-022-01336-w

**Published:** 2022-02-10

**Authors:** Tomasz Kiełczawa, Paweł Sokołowski, Aleksandra Małachowska

**Affiliations:** Faculty of Mechanical Engineering, Wroclaw University of Science and Technology, ul. Łukasiewicza 5, 50-371 Wrocław, Poland

**Keywords:** build-up mechanism, laser ablation, microtexturization, plasma stream modelling, substrate topography, thermal barrier coatings

## Abstract

This study deals with the numerical analysis of the plasma jet behavior close to the substrate surface depending on its topography. It uses a 2D axisymmetric time-dependent CFD model solved with the Ansys Fluent 2020/R1 package. The model takes into consideration the nonlinear thermophysical properties and turbulent phenomena of the plasma jet as well as its interaction with the microtextured substrate. Representative substrate topographies were considered as a boundary condition in the numerical simulations. They correspond to the bond coats used in Thermal Barrier Coating technology, actually APS sprayed NiCrAlY coatings which were experimentally microtextured using various laser unit operational conditions resulting in different substrate topographies. The numerical calculations showed that the substrate topography, modified and controlled in this work by microtexturing, disturbs the homogeneity of the pressure field in the substrate boundary layer resulting in the periodical pressure fluctuation. It was also observed that the relative local pressure disturbance is more significant in the substrate outer regions than close to the centerline. Then, based on the results of numerical calculations, the potential movement of feedstock particles near to the substrate was discussed. It was concluded that the deposition of fine powders, characterized by a low Stokes number, will be influenced by the pressure field distribution near to the substrate and will take place mainly in the local high-pressure zones. Furthermore, the local swirl of plasma taking place in each fine microtexture, created here by laser ablation, privileges the deposition of such particles on the surface asperities. These observations show that the CFD code modeling opens the possibility of predicting the movement and deposition of particles during plasma spraying, which is essential for understanding coating deposition mechanisms in suspension plasma spray.

## Introduction

Over the last decades, thermal barrier coating (TBC) has been a dynamically developed protective coating system for aircraft gas turbines (Ref 1). It allows increasing the operating temperature and lifetime of jet engine components, thus reducing the fuel consumption and the emission of harmful substances into the atmosphere. The further development of thermally sprayed TBCs is focused on two routes: (i) novel spraying technologies or spraying approaches (SPS, SPPS, hybrid spraying, laser or ion beam postprocessing, etc.), and (ii) advanced feedstock materials and microstructures (new materials, functionally graded compositions, multilayer structures, etc.). One of the most promising research areas is the so-called columnar-like TBC. The occurrence of such a structure during thermal spraying is a superposition of several parameters. Firstly, the use of fine powder feedstock is recommended (Ref 2). Secondly, the control over the substrate surface topography opens possibilities to control the TBC build-up mechanism. VanEvery et al. (Ref 3) investigated the influence of the substrate roughness on the probability of the occurrence of a columnar structure. It was proven that the surface roughness performs a role in the formation of the columnar structure as it increases the shadowing effect, which is said to be actively affecting the possibility of a column formation. Bernard et al. (Ref 4) compared two substrates with Ra = 0.6 µm and Ra = 1.5 µm, obtained by sandblasting, in terms of the deposited columns geometry. The study showed that the columns were well developed on the more rough substrate and resulted in a higher overall coating porosity. Consequently, the thermal fatigue performance of such TBCs may be improved as well. Curry et al. (Ref 5) studied the thermal shock resistance of TBCs with as-sprayed, sandblasted, and polished bond coats. The sandblasted and as-sprayed ones showed a much higher cyclic lifetime with nearly 300 cycles to failure compared to the polished bond coats with 150 cycles. Recently, laser pretreatment has been tested in the thermal spraying technology (Ref 6, 7).

Laser microtexturing development opens the possibility of substituting the grid blasting technique or micromachining and obtaining a more uniform and precisely controlled substrate topography, which can be controlled in the micrometer regime and obtained in a very reproducible manner. Lamraoui et al. (Ref 8) investigated the surface laser texturization pretreatment prior to the spraying in terms of increasing the coating adherence in comparison with rough sandblasted surfaces. The surface morphology and mechanical performance were analyzed in the function of the number of shots per an engraved hole. It was observed that the coating toughness can be enhanced by a deep laser pretreatment compared to the reference sandblasted substrates. The idea of omitting the bond coat layer, i.e. bond coatless TBCs with laser-microtextured substrates, was also proposed and such TBCs were investigated in terms of the thermal fatigue performance. It appears that the substrate microtexturization prior to spraying effectively reduces the possibility of top coat delamination after cooling (Ref 9). However, the thermal fatigue performance improvement has also been observed in multilayer TBCs. Khan et al. (Ref 10) investigated the thermal shock resistance of functionally graded YSZ and LaMgAl_11_/YSZ Thermal Barrier Coatings (TBCs). Each functional layer sprayed with the LaMgAl_11_/YSZ coating material was microtextured with a picosecond Nd-YAG laser source with optimized operational parameters to prevent layer recasting. It resulted in 219 thermal cycles before failure for functionally graded TBC microtextured with optimized parameters compared to approximately 70 cycles for as-sprayed conventional YSZ-TBC. In one of our previous works, it was shown that by adjusting the topography parameters via laser microtexturing, a varied morphology of the columnar-like TBCs may be obtained (Ref 6).

The influence of the substrate and substrate surface on the plasma jet and hence the formation of coating is researched usually based on the numerical simulation. Pourang et al. (Ref 11) carried out a numerical analysis of the interaction of the plasma jet with substrates having various geometries, i.e. flat and curved ones. However, the surface roughness was not taken into consideration in those studies. Wang et al. (Ref 12) simulated the TBC build-up mechanism with the Monte Carlo method. This model includes the particle deposition process as the columnar structure of the coating is being build. They also performed a numerical analysis of the plasma stream flow around the sample using the *k*–*ω* SST turbulent model. Both models were 2D and applied separately. Wang et al. (Ref 12) analyzed the process of layer-by-layer growth, while paying attention to the shadowing effect as the cause of the occurrence of the columnar structure and the influence of the roughness, and the sample surface-plasma stream axis angle, on the porosity of the deposited layers. The computational approach of Meillot et al. (Ref 13 considered the ArH_2_ plasma impact on a moving flat substrate, while Jadidi et al. (Ref 14) modeled the interaction between the plasma stream and the flat substrate surface as well as feedstock particles trajectories and statistics in flat substrate boundary layers. Mauer (Ref 15) carried out a numerical analysis of particle trajectories in the substrate boundary layers as well. He proved that the particle Stokes number determines the followed trajectory resulting in a shallow particle impact and lateral columnar structure development for low Stokes number particles. A different approach to modeling the fluid flow in the textured surface boundary layer was taken by Bai et al. (Ref 16), who analyzed the water fluid flow in textured substrate boundary layers on a macro-scale, with 30 mm groove width. They studied the dependence of drag and friction coefficients on the surface texture, V-shaped, saw-tooth, rectangular, and semicircular. It was proven that the surface texturization reduces the drag coefficient in the substrate boundary layer and stabilizes the near-wall flow field.

In this study, the interaction between the plasma jet and the substrate was studied by using numerical approach. The research was mainly oriented on the plasma flow in the substrate boundary layer but considering the specific topographies of the substrate. The phenomena occurring in the substrate proximity are crucial for obtaining a fine and regular columnar-like structure. Taking into consideration the current knowledge discussed in this paragraph such modeling may provide a new insight into the particle deposition and coating build-up mechanisms in plasma spraying, mainly liquid feedstock plasma spraying. The specific topographies were obtained by laser microtexturing and then were mapped and introduced into the numerical model. Then, the plasma flow was modeled and the plasma jet interaction with a microtextured substrate was discussed in the context of obtaining tailored columnar-structured TBCs by using controlled substrate topography.

## Methods of Modeling and Experimental Reference

### Surface Pretreatment

In this work, the laser ablation technique was used as a tool for obtaining an experimental reference of the substrate topography, which was then subjected to numerical modeling.

**Fig. 1:**
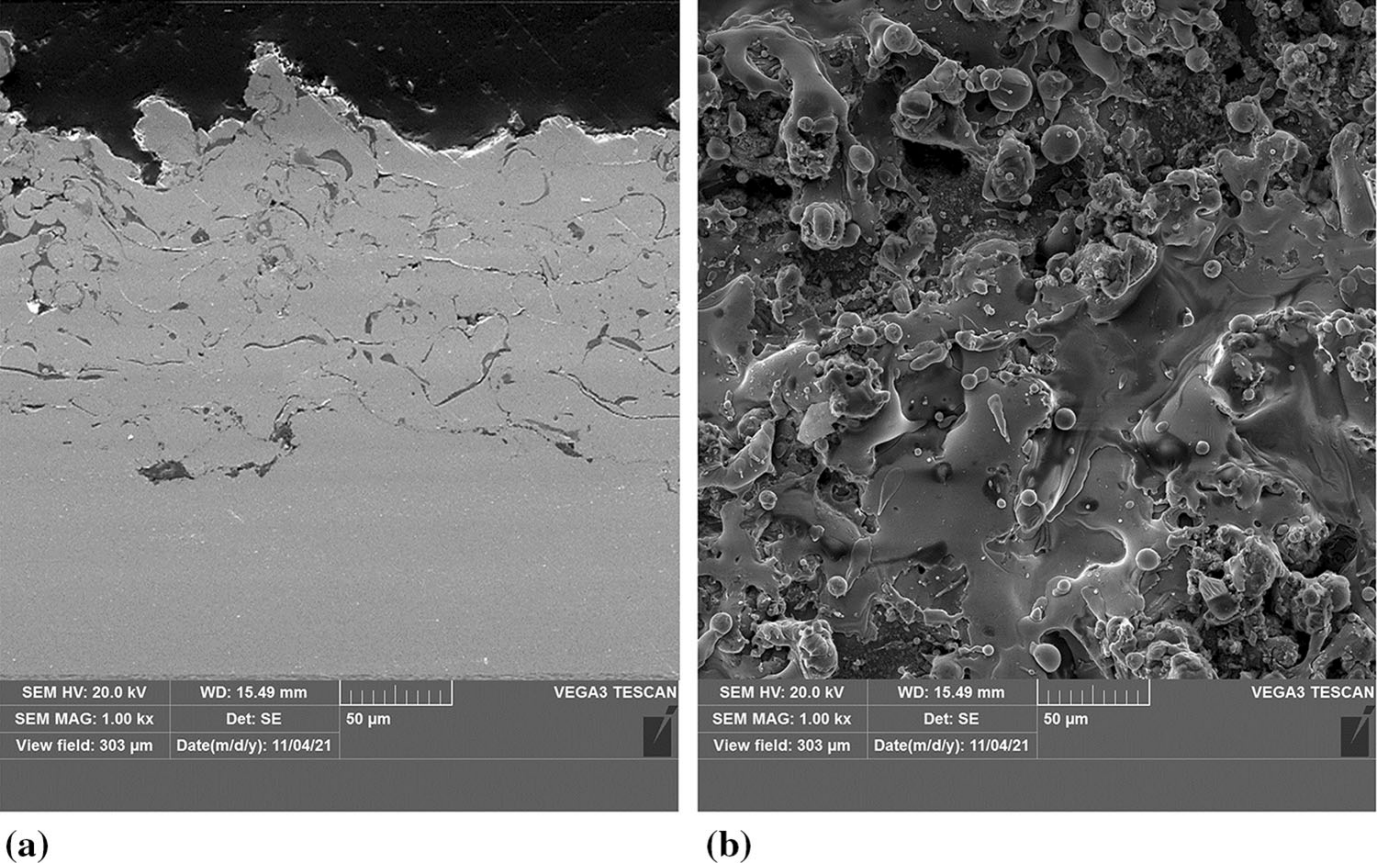
The representative NiCrAlY bond coat, (a) cross-section and (b) surface

As laser pretreatment is considered for the development of the TBC technology, microtexturization was performed on the NiCrAlY bond coat layer, referring to the potential application. The 90-100 µm thick bond coat was sprayed by the means of Atmospheric Plasma Spraying (APS) with the commercially available − 90 + 45 µm AMDRY 963 powder (OC Oerlikon, Freienbach, Switzerland). The cross-section and surface of the as-sprayed bond coat is presented in Fig. 1(a) and (b), respectively. The Ra roughness of such an as-sprayed bond coat, investigated by stylus profilometer MarSurf PS 10 (Mahr, Göttingen, Germany), was approximately 12.8 µm.

**Fig. 2:**
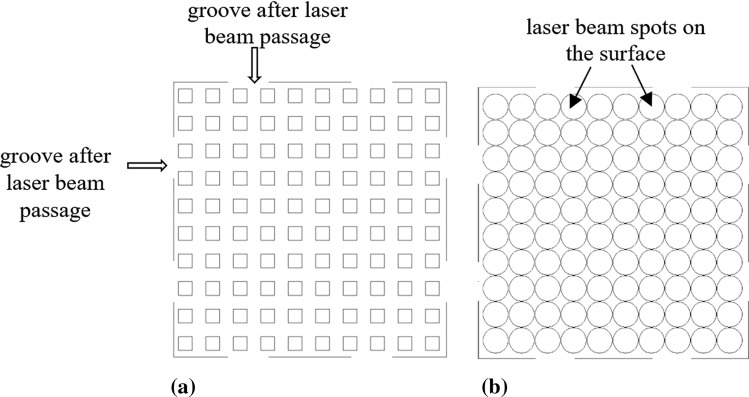
The microtextured substrate topography scheme: (a) groove pattern, (b) hole pattern

Then, the Coherent F20 Varia (Coherent, Santa Clara, USA) laser source with galvo scanner optics was used for microtexturization because it was found to be capable of creating a homogeneous microtexture with an opening starting from ca. 20 micrometers. Based on the recent own experimental approach to this study (Ref 6), a ‘denser’ substrate topography ensures a fine, homogenous, and continuous TBC columnar-like structure. The impacting particle size is another important issue to consider while selecting the groove cross-section. It is essential for both, computational modeling presented in this study, and real application, which will come later. Considering further steps, the topography is considered in the context of Suspension Plasma Sprayed Thermal Barrier Coatings. The typical impinging particles in SPS TBCs are sub-micrometer ones (Ref 17), with a mean powder particle size usually being in the range of 0.5 to 1.5 µm. This is roughly ten times lower than the groove width and depth. Consequently, the coating-forming material may easily enter the groove and be deposited on the groove walls. As the groove geometry is another important variable, the two kinds of preliminary selected patterns were considered, namely the groove pattern and the holes pattern (Fig. 2). Considering such conditions, the laser source operational conditions were optimized to obtain uniform, repeatable microtextures on the as- sprayed NiCrAlY bond coat layer.

**Fig. 3:**
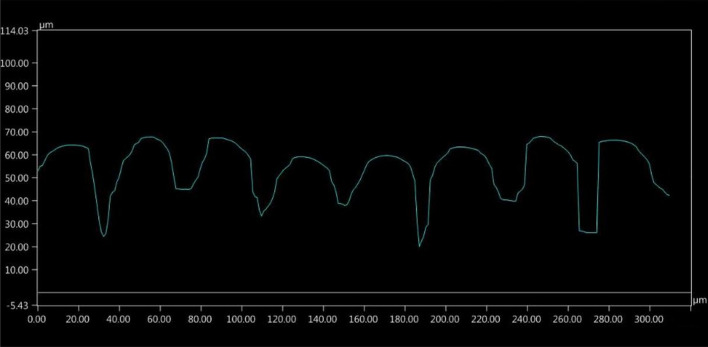
So-called semicircular profile of the substrate microtextured with Case 1 laser source operational conditions

**Fig. 4:**
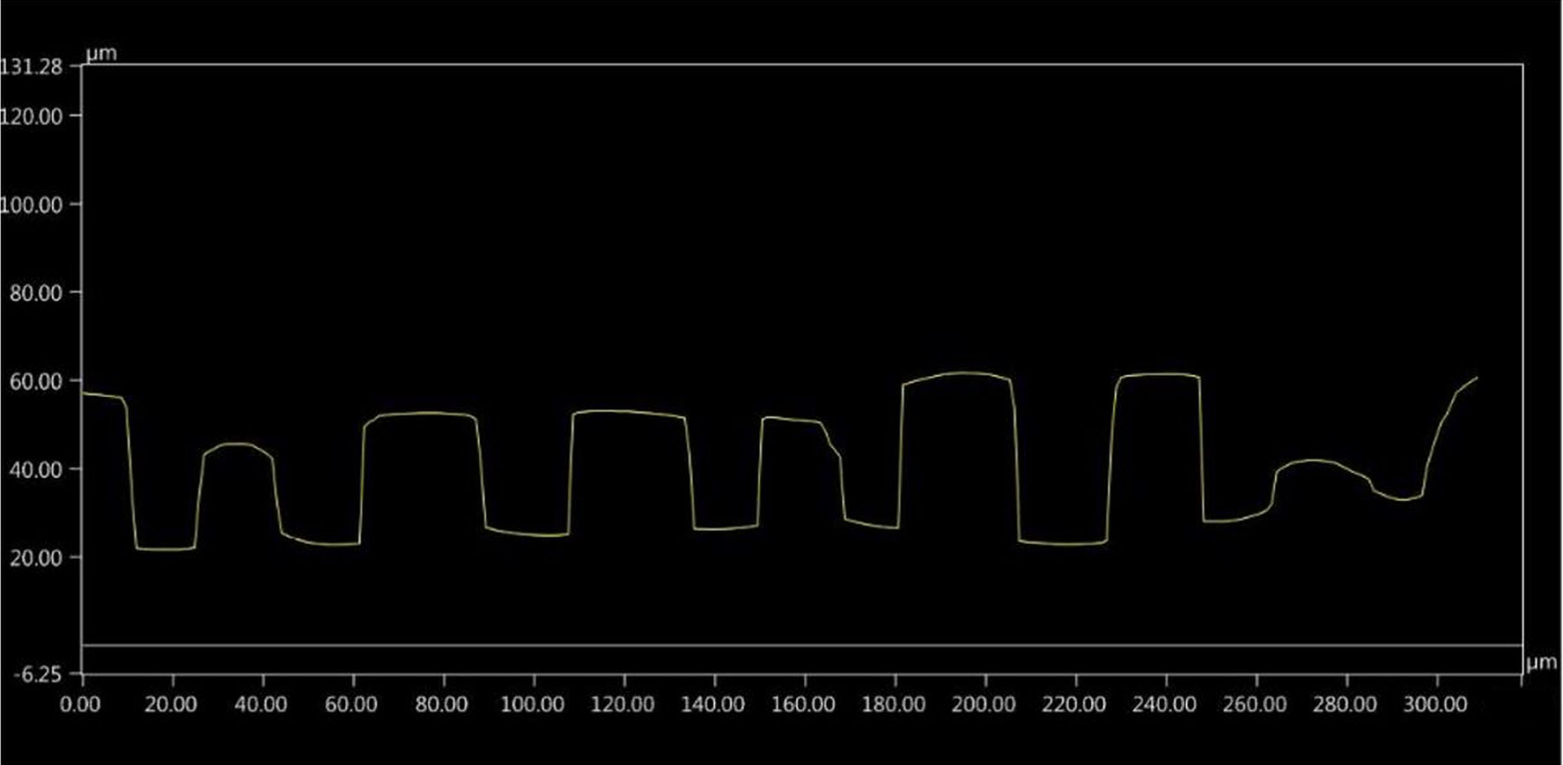
So-called rectangular profile of the substrate microtextured with Case 2 laser source operational conditions

**Fig. 5:**
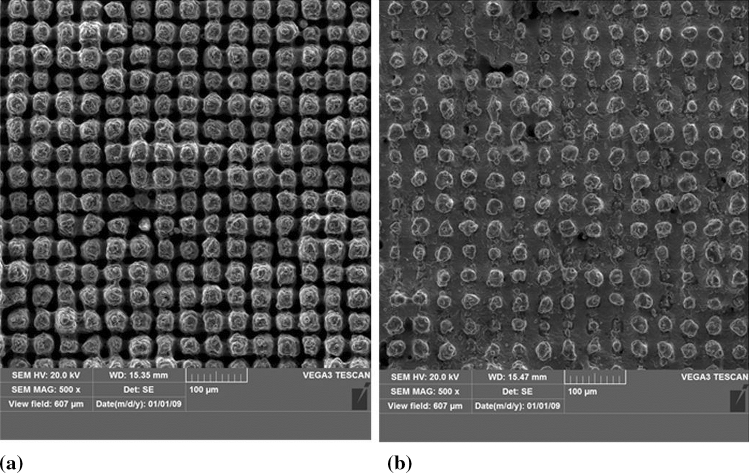
Microtextured substrate topography obtained with, (a) Case 1 and (b) Case 2 laser operational conditions

Then, after laser microtexturing, the samples were observed under a digital microscope Keyence VHX-6000 (Keyence, Osaka, Japan) with motorized *X-*, *Y-*, and *Z*-axes as well as by using scanning electron microscopy (Vega 3, Tescan Orsay Holding, Brno-Kohoutovice, Czech Republic). The topography consisted of grooves or holes with the approximate depth of 20 µm and various profile shapes depending on the laser source operational conditions. Based on a microscopic inspection of laser pretreated substrates, including an in-depth analysis, two types of substrate profiles were selected for further investigation: the so-called semicircular and the so-called rectangular (Fig. 3 and 4, respectively). The SEM micrographs showing the surfaces of these two laser pretreated bond coats are presented in Fig. 5, while the corresponding laser operational parameters are summarized in Table 1.

**Table 1:** Laser source operational conditions

Laser operational conditions	Resulting substrate profile	
	Semicircular	Rectangular
	Case 1	Case 2
Output power, W	12	12
Pulse repetition rate, Hz	50,000	100,000
Texturing speed, mm/s	500	250
Groove spacing (s), µm	40	40
No. of texturization repetitions	7	7

### Topography Modeling and Computational Domain

**Fig. 6:**
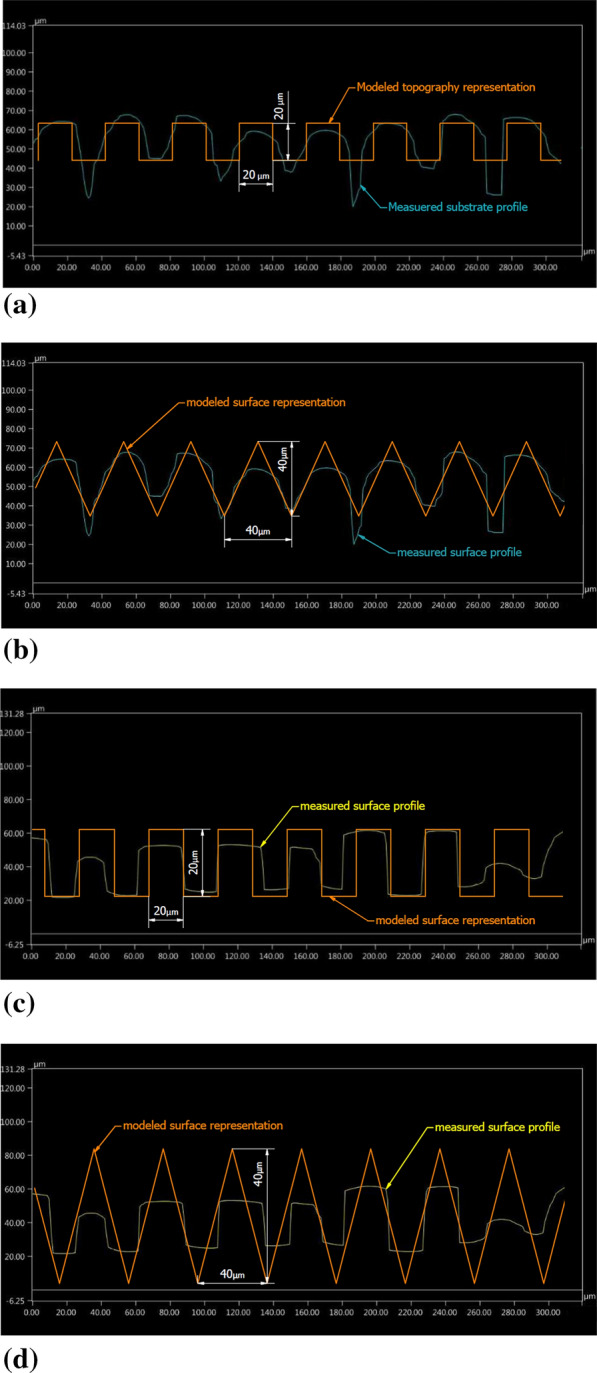
Comparison of the measured substrate profile and modeled substrate representation:

**Fig. 7:**
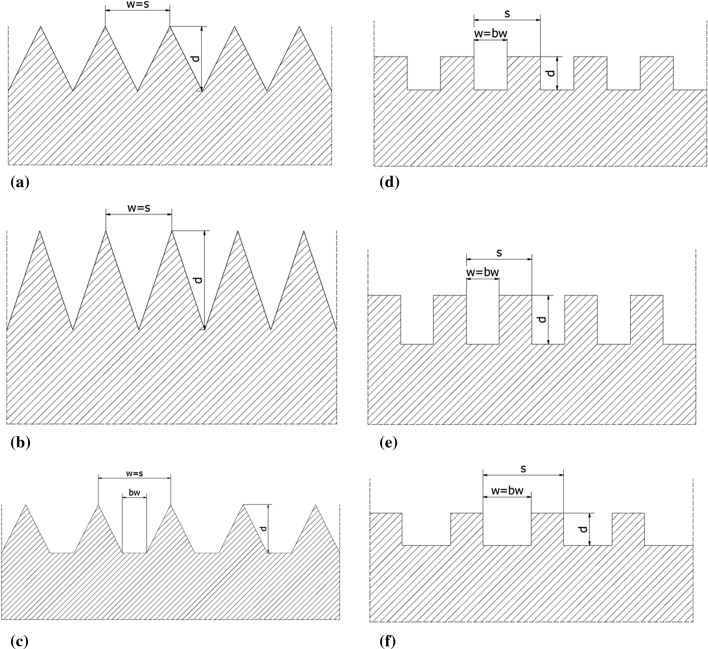
Draft of the outlines of the modeled substrate topography representation variants: (a) SAW 04 V-shape variant, (b) SAW D06 V-shape variant, (c) SAW W06 V-shape variant, (d) REC 02 rectangular variant, (e) REC D03 rectangular variant, (f) DEC W03 rectangular variant

The main interest of this work is the influence of substrate microtexturization on the plasma stream behavior in substrate boundary layers. After the laser pretreatment of NiCrAlY bond coat, two types of substrate topography were selected, resulting in two types of substrate surface representations to be modeled. Figure 6 shows the modeled surface representations sketched over the laser microtextured substrate profiles. The as-produced by laser microtexturing semicircular and rectangular topographies were approximated with, respectively, V-shape and rectangular models considering the best possible conformity of the corresponding profiles. Each mentioned representation was modeled in three variants differing in the groove depth ‘*d*’, width ‘*w*’, spacing ‘*s*’, and the groove bottom width ‘*bw*’. This was done to determine the influence of the mentioned geometrical parameters on the plasma stream characteristics in contact with the microtextured surface. Table 2 shows the list of all modeled variants and Fig. 7 shows the outlines of the respective profiles.

**Table 2:** List of the modeled substrate surface representations

Modeled surface representation	Surface profile type	Modeled surface representation type	*d*, µm	*w*, µm	*s*, µm	*bw*, µm	Modeled profile geometry	
SAW_04	Semicircular	V-shape	40	40	40	0	Figure 7	a
SAW_D06	Semicircular	V-shape	60	40	40	0		b
SAW_W06	Semicircular	V-shape	40	60	60	20		c
REC_02	Rectangular	Rectangular	20	20	40	20		d
REC_D03	Rectangular	Rectangular	30	20	40	20		e
REC_W03	Rectangular	Rectangular	20	30	50	30		f

*d*, groove depth; *w*, groove width (measured at the top of the groove); *s*, beam pass spacing; *bw*, groove bottom width

**Fig. 8:**
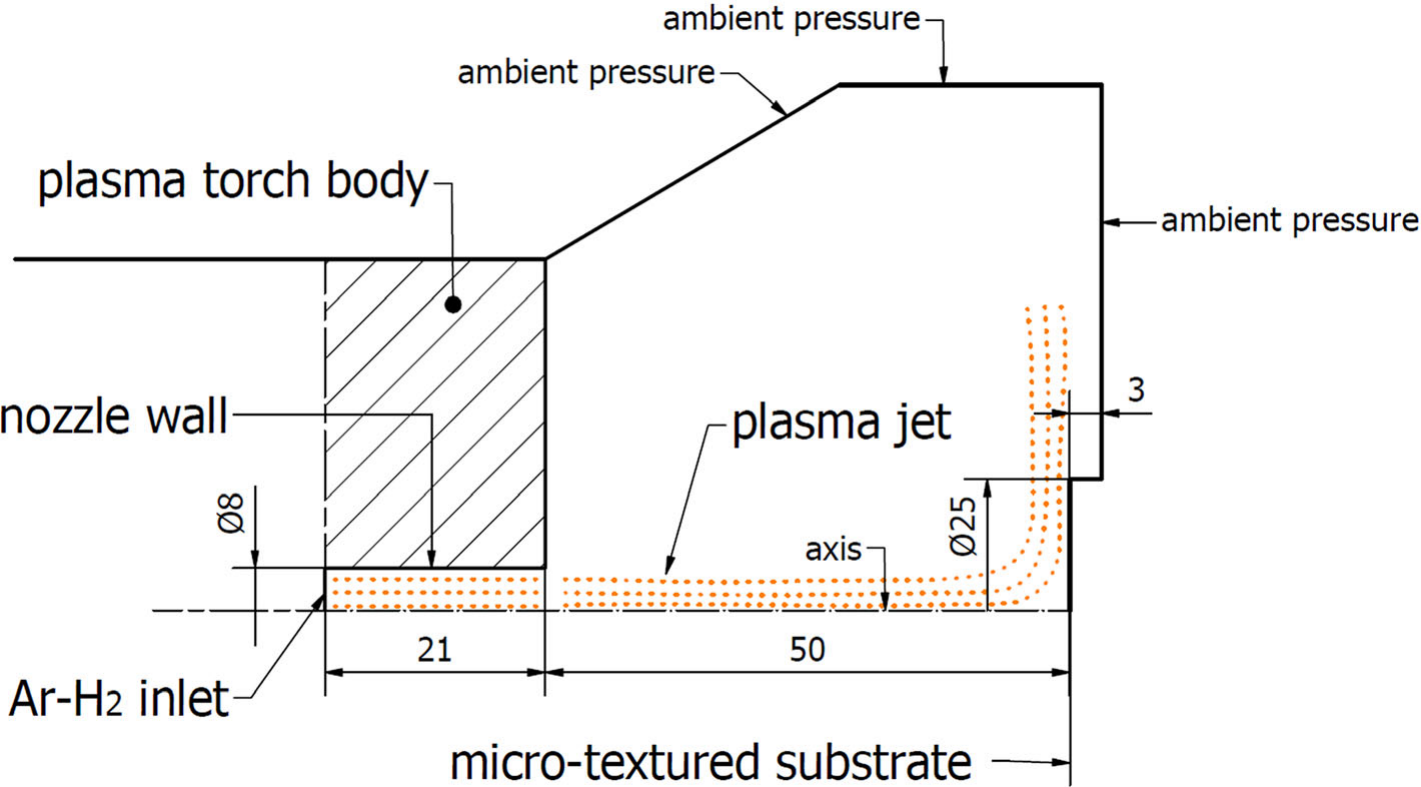
Computational domain draft

Apart from the fine-modeled substrate boundary layer, the geometric model considered the free-jet area between the substrate and the SG-100 (Praxair, Indianapolis, US) torch nozzle. The anode with a length of 21 mm and a nozzle with a diameter of 8 mm were modeled. The diameter of the SG-100 torch front cover was 67 mm. The sample surface was located 50 mm from the nozzle outlet. A cylindrical sample substrate with a diameter of 25 mm and a thickness of 3 mm were considered in all the mentioned surface topography variants. The computational domain, as a whole, is presented in Fig. 8. Consequently, the very fine 2D axisymmetric discrete model consisted of 6 million structured QUAD4 type cells. Additionally, the substrate wall-adjacent mesh was locally refined to capture the plasma stream flow inside the grooves. It is also needed due to the high velocity gradients occurring in the substrate boundary layers.

### Plasma Jet Mathematical Modeling

Modeled plasma medium was considered as a single phase Newtonian compressible mixture of chemically inert gases defined by species mixing laws. The time-dependent calculations were done using the Pressure-Based solver, axisymmetric 2D discrete model and PISO scheme for Pressure-Velocity Coupling due to its better stability under porous jump boundary conditions. Although coupled schemes offer a more efficient single phase implementation for steady-state flows, those methods may suffer from convergence issues in specific flow configurations. This is explained is detail in the section *Choosing the Pressure–Velocity Coupling Method Ansys Fluent* of *Ansys Fluent User Guide* (Ref 18).

The mass conservation for the 2D axisymmetric problem is solved as:

1 $$\frac{\partial \rho }{{\partial t}} + \frac{\partial }{\partial x}\left( {\rho \overrightarrow {{v_{x} }} } \right) + \frac{\partial }{\partial r}\left( {\rho v_{r} } \right) + \frac{{\partial v_{r} }}{r} = S_{m} ,$$

where *ρ* is the density of the gas mixture, *x* is the axial coordinate, r is the radial coordinate, *v*_*x*_ and *v*_r_ are axial and radial velocity, respectively. *S*_m_ is the mass source of the dispersed phase such as droplets of the evaporated liquid.

The axial and radial momentum conservation equations are:

2 $$\begin{aligned} & \frac{\partial }{\partial t}\left( {\rho v_{x} } \right) + \frac{1}{r}\frac{\partial }{\partial r}\left( {r\rho v_{x} v_{x} } \right) + \frac{1}{r}\frac{\partial }{\partial r}\left( {r\rho v_{r} v_{x} } \right) = - \frac{\partial p}{{\partial r}} + \frac{1}{r}\frac{\partial }{\partial x}\left[ {r\mu \left( {2\frac{{\partial v_{x} }}{\partial x} - \frac{2}{3}\left( {\nabla \cdot \vec{v}} \right)} \right)} \right] \\ & \quad + \frac{1}{r}\frac{\partial }{\partial r}\left[ {r\mu \left( {\frac{{\partial v_{x} }}{\partial r} + \frac{{\partial v_{r} }}{\partial x}} \right)} \right] + F_{x} \\ \end{aligned}$$

and

3 $$\begin{aligned} & \frac{\partial }{\partial t}\left( {\rho v_{{\text{r}}} } \right) + \frac{1}{r}\frac{\partial }{\partial x}\left( {r\rho v_{x} v_{{\text{r}}} } \right) + \frac{1}{r}\frac{\partial }{\partial r}\left( {r\rho v_{r} v_{{\text{r}}} } \right) = - \frac{\partial p}{{\partial r}} + \frac{1}{r}\frac{\partial }{\partial x}\left[ {r\mu \left( {\frac{{\partial v_{{\text{r}}} }}{\partial x} + \frac{{\partial v_{x} }}{\partial r}} \right)} \right] \\ & \quad + \frac{1}{r}\frac{\partial }{\partial r}\left[ {r\mu \left( {2\frac{{\partial v_{{\text{r}}} }}{\partial r} - \frac{2}{3}\left( {\nabla \cdot \vec{v}} \right)} \right)} \right] \\ & \quad - 2\mu \frac{{v_{{\text{r}}} }}{{r^{2} }} + \frac{2}{3}\frac{\mu }{r}\left( {\nabla \cdot \vec{v}} \right) + \rho \frac{{v_{z}^{2} }}{r} + F_{{\text{r}}} \\ \end{aligned}$$

respectively, where:

4 $$\nabla \cdot \vec{v} = \frac{{\partial v_{x} }}{\partial x} + \frac{{\partial v_{{\text{r}}} }}{\partial r} + \frac{{v_{{\text{r}}} }}{r},$$

*v*_*z*_ is the swirl velocity (Ref 19), μ is the molecular viscosity, *p* is the static pressure, and *F*_*x*_ and *F*_r_ are the axial and radial components of the external body forces, respectively.

This was applied to the Energy Equations with the Diffusion Energy Source model included. Based on the SG-100 plasma torch operating condition (see Table 3) and the nozzle geometry, the volumetric heat source with the energy efficiency was calculated from (Ref 20):

5 $$P_{{{\text{gun}}}} = \frac{{\eta_{{\text{t}}} }}{V} E\left( t \right) \times I,$$

**Table 3:** Plasma spray boundary conditions

Plasma gun operating conditions	
Nozzle diameter, mm	8
Arc current, A	800
Arc voltage, V	40
Thermal efficiency, %	47
Ar-H_2_ mass flow rate, g/s	1.48
Powder feed rate, g/min	20

Boundary conditions	
Type	Mass flow inlet
*Plasma gas inlet*	
Plasma gas composition	Ar—45 slpm
	H_2_—5 slpm
Turbulence intensity, %	5
Hydraulic diameter, mm	8
Plasma gas temperature, K	300

Type	Pressure inlet
*Ambient pressure inlet*	
Ambient gas composition	N_2_—80 % vol.
	O_2_—20% vol.
Ambient temperature, K	300
Turbulence intensity, %	5
Viscosity Ratio	10

Type	Stationary wall
*Substrate wall*	
Wall temperature, K	300
Wall thickness, mm	3
Heat flux, W/m^2^	0
Heat generation, W/m^3^	0

where *P*_gun_ is the energy generated by the SG-100 plasma gun, *E* is the arc voltage, *I* is the current, and *V* is the volume of the gun nozzle. Thermal efficiency *η*_t_ of 47% was assumed based on Farrokhpanah et al. (Ref 21) studies, as they used the same plasma torch type and nozzle geometry. The properties of the plasma were taken into consideration by modifying the plasma gases characteristics. Highly nonlinear thermophysical properties of the Ar-H_2_ plasma were included in the calculations based on the work of Boulos et al. (Ref 22), who determined all the needed plasma properties in the full range of plasma parameters generated by the SG-100 torch.

The plasma gas inlet, ambient pressure inlet, and substrate wall boundary conditions were set according to the torch operational conditions (Table 3). On top of that, the simulation duration time was optimized and set as 0.05 s to obtain the converged calculation with a quasi-constant outlet mass-flow and possibly reduced calculation cost. Additionally, in such a short-term simulation the heat transfer to the substrate is very limited and the heat equations for the substrate were not considered here. In this work, the plasma flow behavior in the substrate boundary layer has been analyzed in terms of the field variable gradients and the direction of the flow vectors, which are affected by turbulent phenomena. The wall-function was defined as for *k*–*ω* models, where the *ω* value at the wall is:

6 $$\omega_{{\text{w}}} = \frac{{\rho \left( {u^{*} } \right)^{2} }}{\mu }\omega^{ + },$$

where:

7 $$\omega^{ + } = \frac{6}{{\beta_{i} \left( {y^{ + } } \right)^{2} }}$$

for the laminal sublayer and

8 $$\omega^{ + } = \frac{1}{{\sqrt {\beta_{\infty }^{*} } }}\frac{{{\text{d}}u_{{{\text{turb}}}}^{ + } }}{{{\text{d}}y^{ + } }}$$

for the logarithmic region. *β*_*i*_ and β^*^ are closure coefficients, *y*^+^ is the dimensionless distance from the wall surface, u* and *u*^+^_turb_ are *k*–*ω* model velocity variables.

This was taken into consideration with the use of two mathematical models: the Shear Stress Transport (SST) model and the Reynolds Stress Model (RSM). The SST Transient model was used only for the initial calculations and as a control reference due to the lower computational cost. For fine calculations, RSM was used due to a better vortex mapping in the plasma stream volume. The transport equations of RSM are as follows:

9 $$\begin{aligned} & \frac{\partial }{\partial t}\left( {\rho \overline{{u_{i}^{\prime } u_{j}^{\prime } }} } \right) + \frac{\partial }{{\partial x_{k} }}\left( {\rho u_{k} \overline{{u_{i}^{\prime } u_{j}^{\prime } }} } \right) = - \frac{\partial }{{\partial x_{k} }}\left[ {\rho \overline{{u_{i}^{\prime } u_{j}^{\prime } u_{k}^{\prime } }} + \overline{{p^{\prime } \left( {\delta_{kj} u_{i}^{\prime } + \delta_{ik} u_{j}^{\prime } } \right)}} } \right] + \frac{\partial }{{\partial x_{k} }}\left[ {\mu \frac{\partial }{{\partial x_{k} }}\left( {\overline{{u_{i}^{\prime } u_{j}^{\prime } }} } \right)} \right] \\ & \quad - \rho \left( {\overline{{u_{i}^{\prime } u_{k}^{\prime } }} \frac{{\partial u_{j} }}{{\partial x_{k} }} + \overline{{u_{j}^{\prime } u_{k}^{\prime } }} \frac{{\partial u_{i} }}{{\partial x_{k} }}} \right) - \rho \beta \left( {g_{i} \overline{{u_{j}^{\prime } \theta }} + g_{j} \overline{{u_{i}^{\prime } \theta }} } \right) + p^{\prime } \left( {\frac{{\partial u_{i}^{\prime } }}{{\partial x_{j} }} + \frac{{\partial u_{j}^{\prime } }}{{\partial x_{i} }}} \right) \\ & \quad - 2\mu \frac{{\partial u_{i}^{\prime } }}{{\partial x_{k} }}\frac{{\partial u_{j}^{\prime } }}{{\partial x_{k} }} - 2\rho \Omega_{k} \left( {\overline{{u_{j}^{\prime } u_{m}^{\prime } }} \varepsilon_{ikm} + \overline{{u_{i}^{\prime } u_{m}^{\prime } }} \varepsilon_{ikm} } \right) + S_{{{\text{user}}}} \\ \end{aligned}$$

where:

$\frac{\partial }{\partial t}\left( {\rho \overline{{u_{i}^{\prime } u_{j}^{\prime } }} } \right)$—local time derivative, $\frac{\partial }{{\partial x_{k} }}\left( {\rho u_{k} \overline{{u_{i}^{\prime } u_{j}^{\prime } }} } \right) = C_{ij}$—convection, $- \frac{\partial }{{\partial x_{k} }}\left[ {\rho \overline{{u_{i}^{\prime } u_{j}^{\prime } u_{k}^{\prime } }} + \overline{{p^{\prime } \left( {\delta_{kj} u_{i}^{\prime } + \delta_{ik} u_{j}^{\prime } } \right)}} } \right] = D_{T,ij}$—turbulent diffusion, $\frac{\partial }{{\partial x_{k} }}\left[ {\mu \frac{\partial }{{\partial x_{k} }}\left( {\overline{{u_{i}^{\prime } u_{j}^{\prime } }} } \right)} \right] = D_{L,ij}$—molecular diffusion, $- \rho \left( {\overline{{u_{i}^{\prime } u_{k}^{\prime } }} \frac{{\partial u_{j} }}{{\partial x_{k} }} + \overline{{u_{j}^{\prime } u_{k}^{\prime } }} \frac{{\partial u_{i} }}{{\partial x_{k} }}} \right) = P_{ij}$—stress production, $- \rho \beta \left( {g_{i} \overline{{u_{j}^{\prime } \theta }} + g_{j} \overline{{u_{i}^{\prime } \theta }} } \right) = G_{ij}$—buoyancy production, $p^{\prime } \left( {\frac{{\partial u_{i}^{\prime } }}{{\partial x_{j} }} + \frac{{\partial u_{j}^{\prime } }}{{\partial x_{i} }}} \right) = \emptyset_{ij}$—pressure strain, $- 2\mu \frac{{\partial u_{i}^{\prime } }}{{\partial x_{k} }}\frac{{\partial u_{j}^{\prime } }}{{\partial x_{k} }} = \varepsilon_{ij}$—dissipation, $- 2\rho \Omega_{k} \left( {\overline{{u_{j}^{\prime } u_{m}^{\prime } }} \varepsilon_{ikm} + \overline{{u_{i}^{\prime } u_{m}^{\prime } }} \varepsilon_{ikm} } \right) = F_{ij}$—production by system rotation, $S_{{{\text{user}}}}$—user-defined Source Term.

## Results and Discussion

### Comparison of Turbulent Flow Models

In general, Eddy Viscosity models are a good compromise between the quality of the boundary layer flow simulation and the high velocity plasma free stream near the domain axis and the stream core. However, the *k*–*ω* Wilcox’ models seem to be oversensitive to the influence of the boundary conditions (Ref 23). On the other hand, the alternative *k*–*ε* models have significant limitations in the simulation of the boundary layer (Ref 24). The highly disordered and turbulent nature of the plasma flow (Ref 14, 25) makes these restrictions even more inconvenient. For these reasons, the RSM model is the most widely used in the case of plasma flow calculations. With RSM, Reynolds stresses are solved directly using the transport equations, avoiding the isotropic viscosity assumption of other models. That opens a window for a highly swirling flow computation with a streamline curvature, accurate mapping of swirl and rotation as well as for capturing large velocity gradients. Although the computational cost is significant, it is still reduced compared to the Large Eddy Simulation (LES) (Ref 25). Another superiority of RSM model over SST *k*–*ω* and LES models has been discussed by Ismail et al. (Ref 26). The open subsonic wind tunnel test chamber was modeled for the comparative analysis of the turbulence models. The results prove that RSM captures the interaction between strain and stress components in the boundary layers which are omitted by the SST *k*–*ω* and LES models (Ref 21). However, RSM requires a relatively large number of equations to be solved in parallel, resulting in the convergence limitation. The dense finite element mesh is also required for accurate swirl mapping and maintaining grid refinement below y* < 5 for the proper wall-functions behavior. It is explained in detail in the Fluent Theory Guide (Ref 19) and the Fluent User’s Guide (Ref 18).

**Fig. 9:**
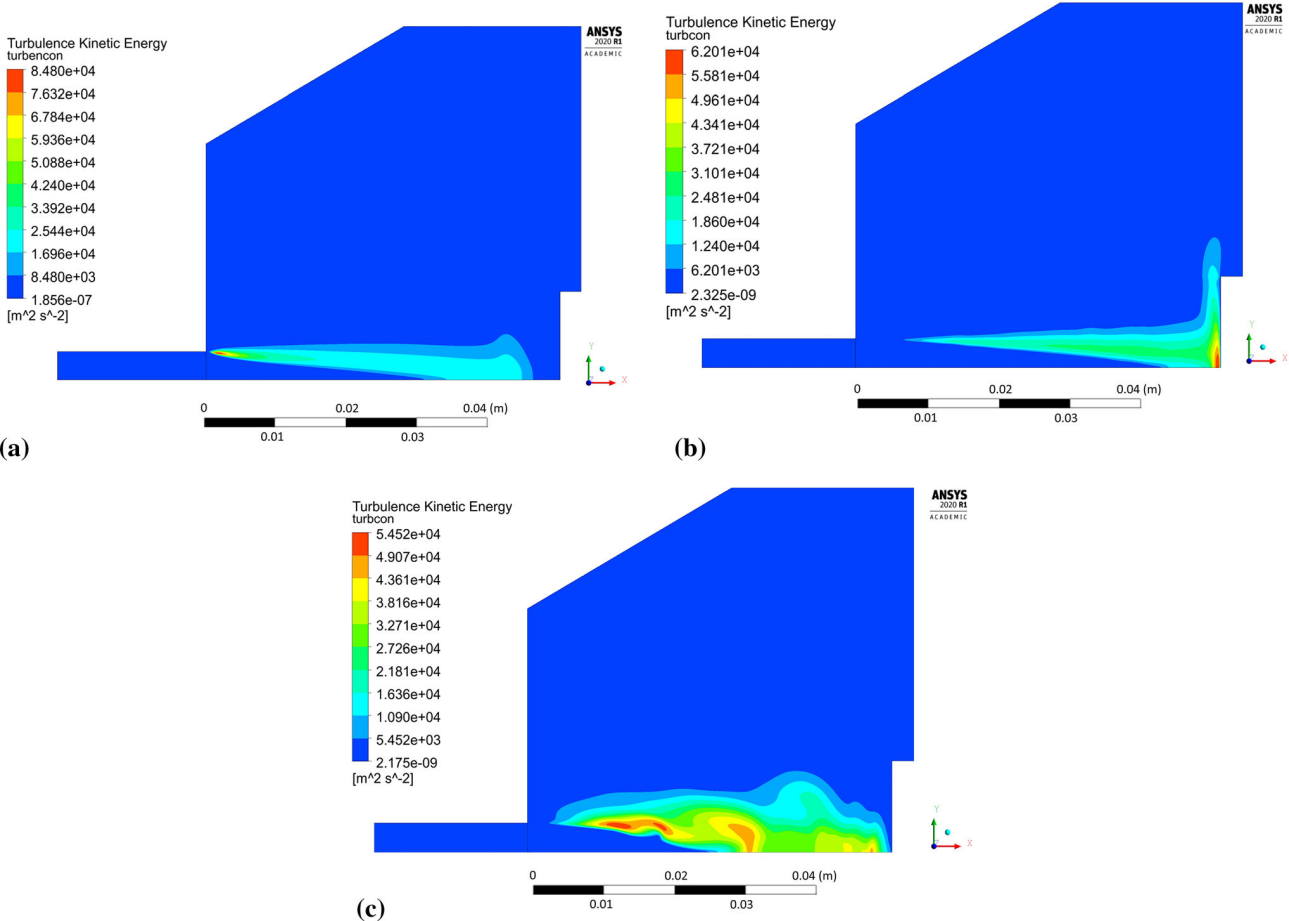
Turbulence energy production bubble generated with: (a) Shear Stress Transport model—quasi-steady state, (b) Reynolds Stress Model—quasi-steady state, (c) Reynolds Stress Model—the moment the plasma jet reaches the substrate surface

As mentioned, theoretical characteristics of turbulent flow mathematical models, such as the SST Transient model and the RSM model, determined the representation of the field of turbulence energy in the modeled plasma stream, both in the free-jet area and the substrate boundary layers. Depending on the mathematical model for turbulence energy generation, different turbulence production bubble geometries were observed. The Shear Stress Transport (transient-SST) model based on Eddy Viscosity Theory generated a concentrated field of turbulence energy near the nozzle edge progressively descending along the jet axis (Fig. 9a). The Reynolds Stress Model (RSM) model resulted in the turbulence energy field ascending along the plasma stream with its maximum in the near-axis area of the sample surface boundary layer (Fig. 9b). Direct solving of the stress tensor components allowed considering the turbulent phenomena in the sample’s near-wall area (Fig. 8). Additionally, RSM allowed observing the turbulence energy filed evolution throughout the plasma stream stabilization process. Fig 9 also compares the unsteady state after 0.001s (Fig. 9a and b) and the quasi-steady state after 0.05s of the simulation (Fig. 9c). Although the influence of the substrate surface topography on the boundary layer flow was investigated in a quasi-steady state, it opens possibilities of analyzing unsteady states using RSM.

### Microtextured Substrate Boundary Layer Analysis

The aim of this study is to understand the influence of specific substrate topographies obtained by laser microtexturing on the plasma flow. Then, the potential mechanism of deposition of coating-forming particles in different plasma spray processes may be analyzed. Here, it was done in the context of further TBC processing by Suspension Plasma Spraying. To achieve this, the simulation was carried out taking into consideration the plasma flow in the entire calculation domain shown in Fig. 8. The use of the RSM model allowed to consider the turbulent phenomena in the sample near-wall layers. This is impossible with simpler Eddy Viscosity models, e.g. SST transient, as previously discussed.

**Fig. 10:**
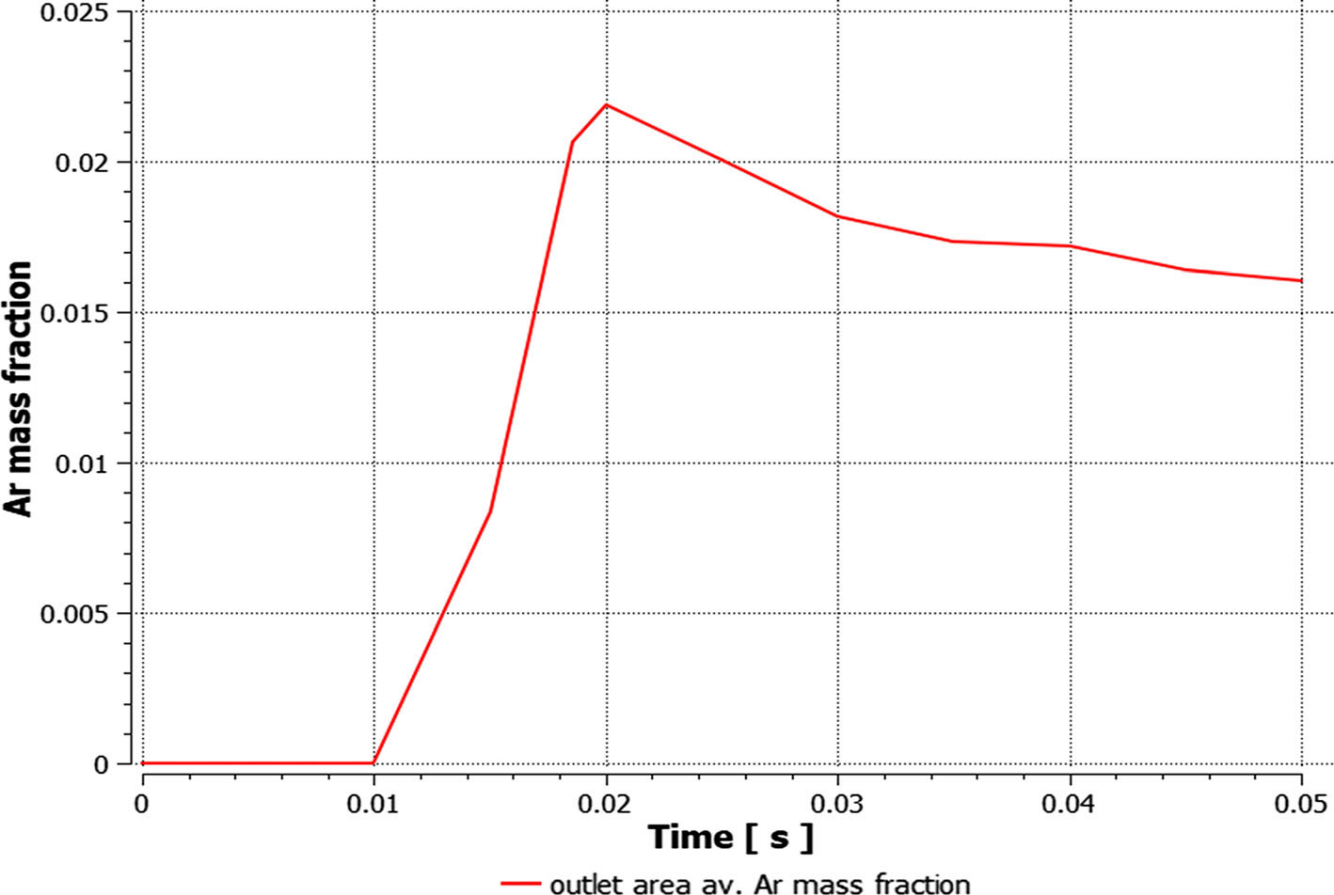
Mass flow of argon plasma component through the computational domain outlet

**Fig. 11:**
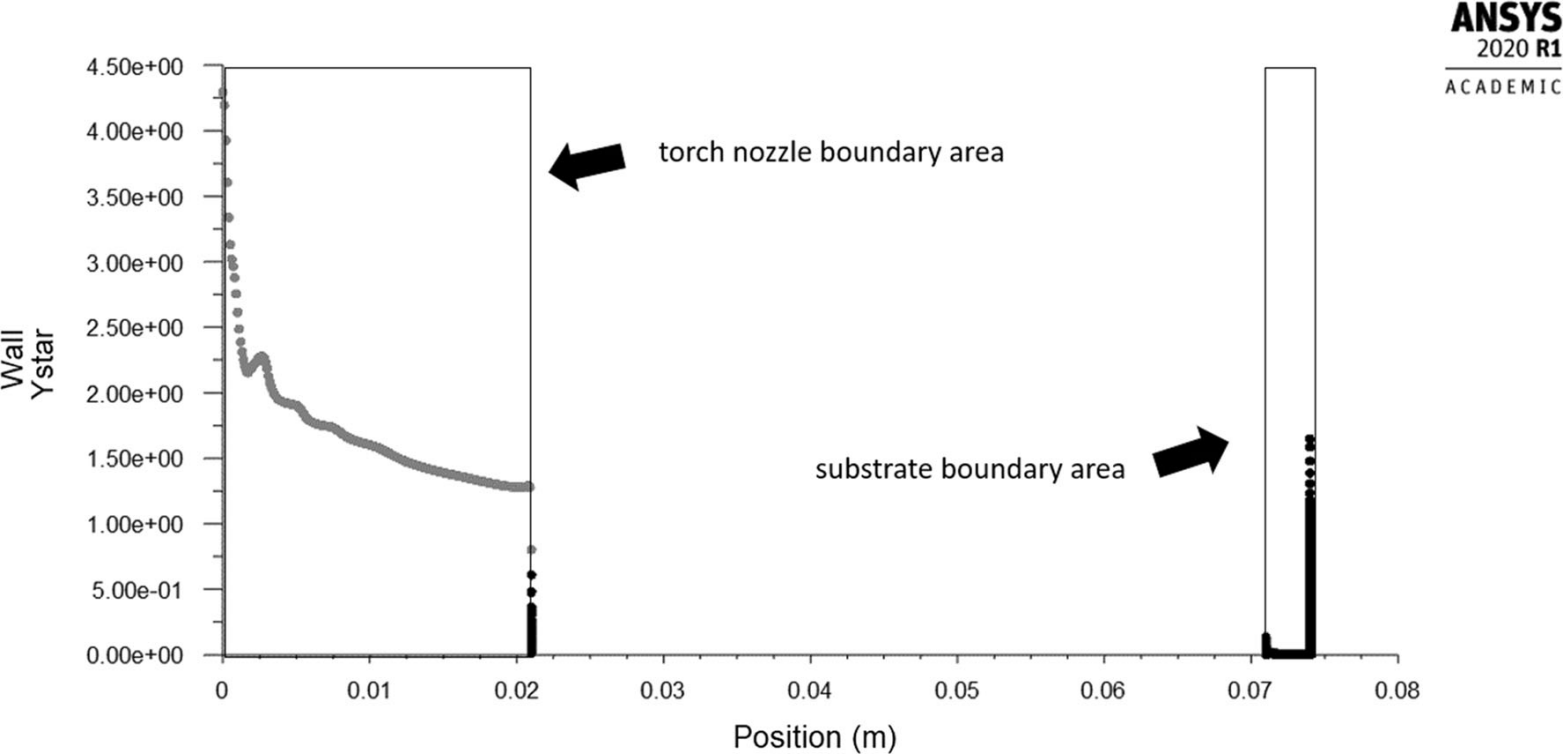
Boundary layer mesh dependent y* values in the computational domain boundary regions

**Fig. 12:**
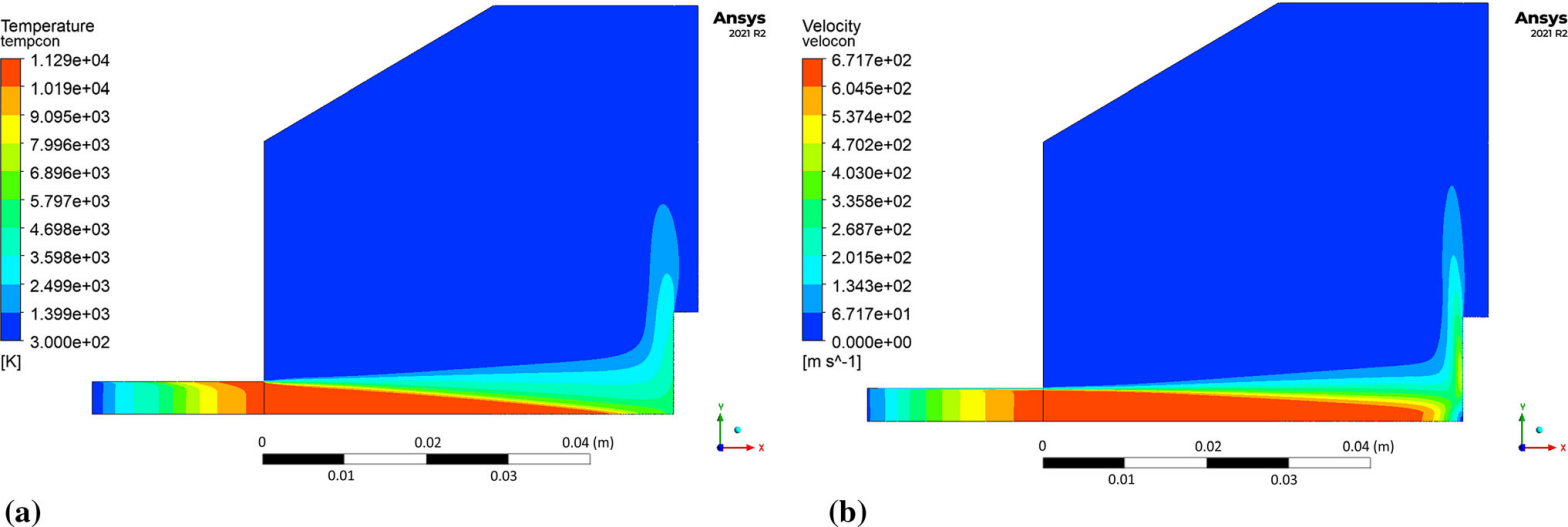
Plasma flow in the computational domain: (a) temperature profile, (b) velocity profile

**Fig. 13:**
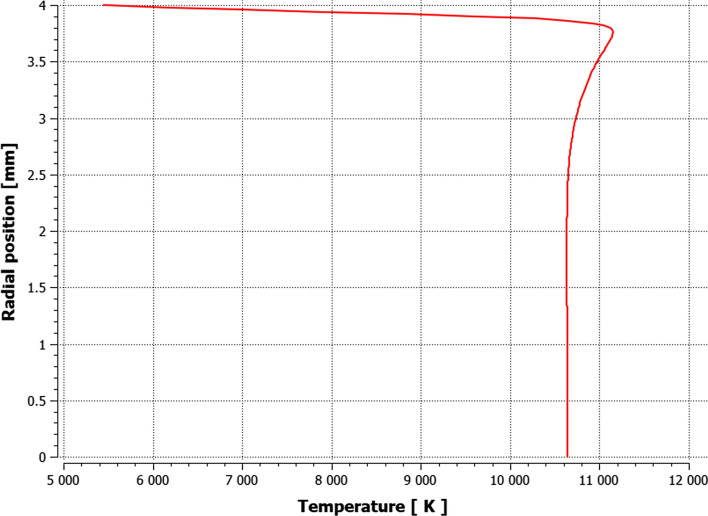
The temperature profile at the SG-100 plasma torch nozzle exit

**Fig. 14:**
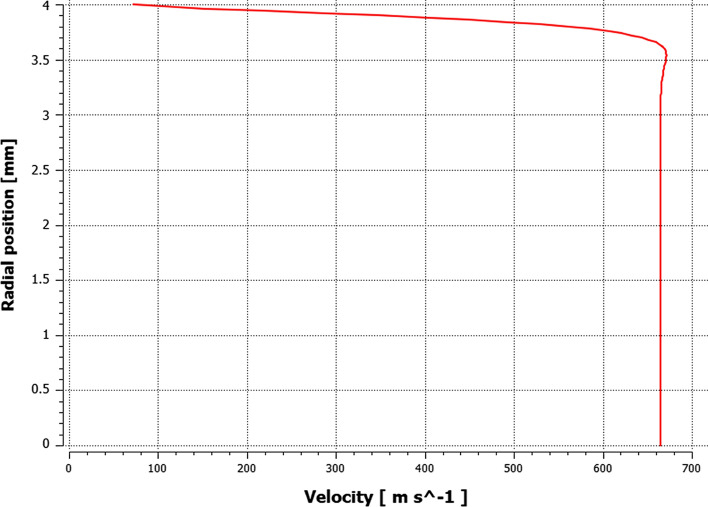
The velocity profile at the SG-100 plasma torch nozzle exit

The influence of the substrate topography on the plasma stream in the boundary layer was analyzed after 0.05 s in a quasi-steady state of converged calculations. After approximately 0.02 s of calculation time, the outlet mass flow of plasma components held a quasi-steady value, which indicates a quasi-steady state of the process (Fig. 10). Furthermore, the mesh-dependent y* variable was analyzed to ensure proper domain conditions for the wall-functions (y* < 5). Fig. 11 shows y* values below 5 in the torch boundary layer, and primarily, in the substrate boundary layers. At this point, the whole domain velocity and temperature field contours were analyzed after 0.05 s for the validation purpose (Fig. 12). Particularly, the temperature and velocity profiles at the nozzle exit were critical (Fig. 13 and 14). The results are highly comparable with the studies focused on the plasma generated with SG-100 and alternative 3MB plasma torch with 6 mm nozzle (Ref 21, 27).

**Fig. 15:**
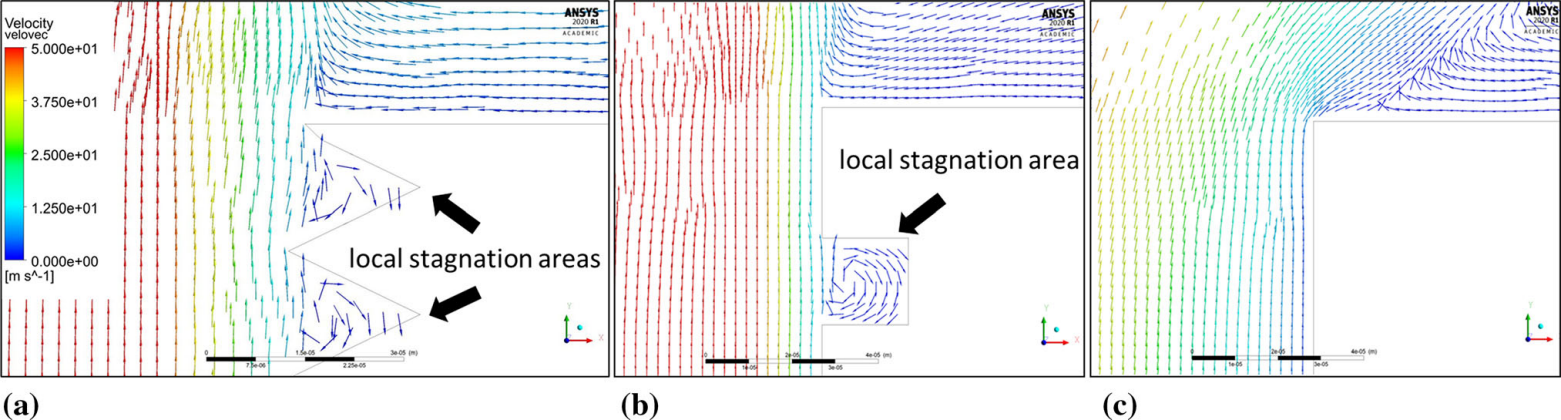
Plasma flow in the substrate boundary layers close to the edge of the substrate, (a) velocity vectors for the microtextured substrate with V-shape grooves (b) velocity vectors for the microtextured substrate with rectangular grooves (c) velocity vectors for the reference flat substrate; (the axial and radial scale is equal)

**Fig. 16:**
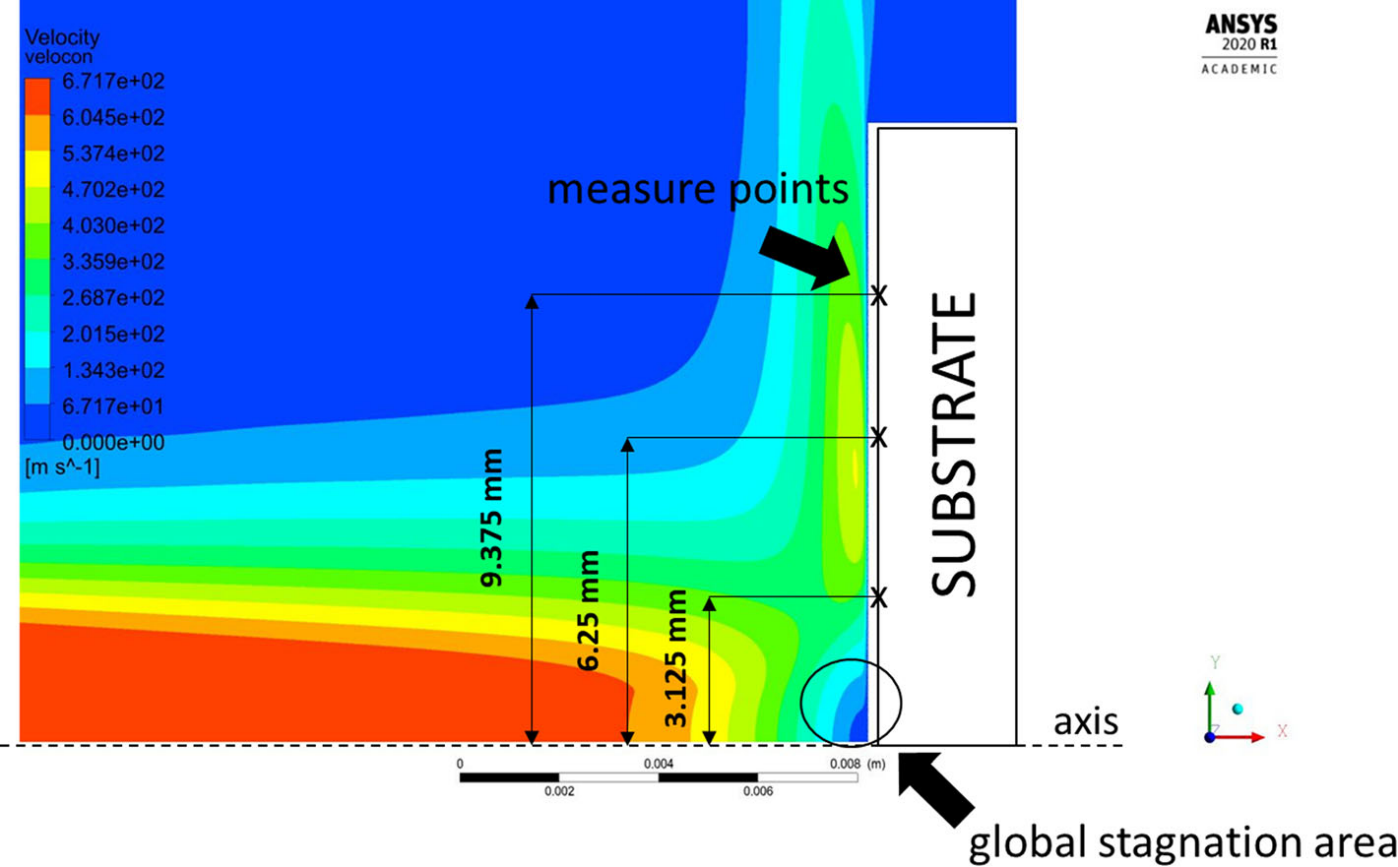
The location of local pressure measure points

The plasma flow direction changes were observed in the boundary layers once the microtextured sample geometry was applied. As the stream reached the edge of the groove, it split into the swirl inside the grooves and the component flowing parallel to the sample surface. The plasma swirled inside the groove with a much lower local velocity than the plasma streamwise velocity. That resulted in the occurrence of local periodical stagnation areas inside the grooves, which is characteristic for every analyzed rectangular and V-shaped topography. Fig. 15 shows the plasma flows for microtextured and flat reference substrate as well as a comparison of the stagnation areas. This phenomenon was investigated by Raayai-Ardakani et al. (Ref 28) in terms of drag reduction in the boundary layers, which is beneficial for the fine feedstock deposition on the substrate asperities. Firstly, the mentioned drag reduction results in pulling the maximum of plasma flow velocity closer to the substrate surface, which can be seen in Fig. 15(a) and (b) especially when compared to the flat substrate shown in Fig. 15(c). Thus, the particles dragged in the plasma stream have also greater kinetic energy (Ref 29). Secondly, the feedstock residence time inside the groove is extended and the overall potential of the coating material deposition on the inner walls of the grooves increases. This phenomenon occurs especially far from the domain axis and the global stagnation area (Fig. 16). Bai (Ref 16) obtained the same effect for the water flow in a comparable scale of substrate topography (30 µm groove width) and using RSM.

### Substrate Boundary Layer Pressure Field Analysis

**Fig. 17:**
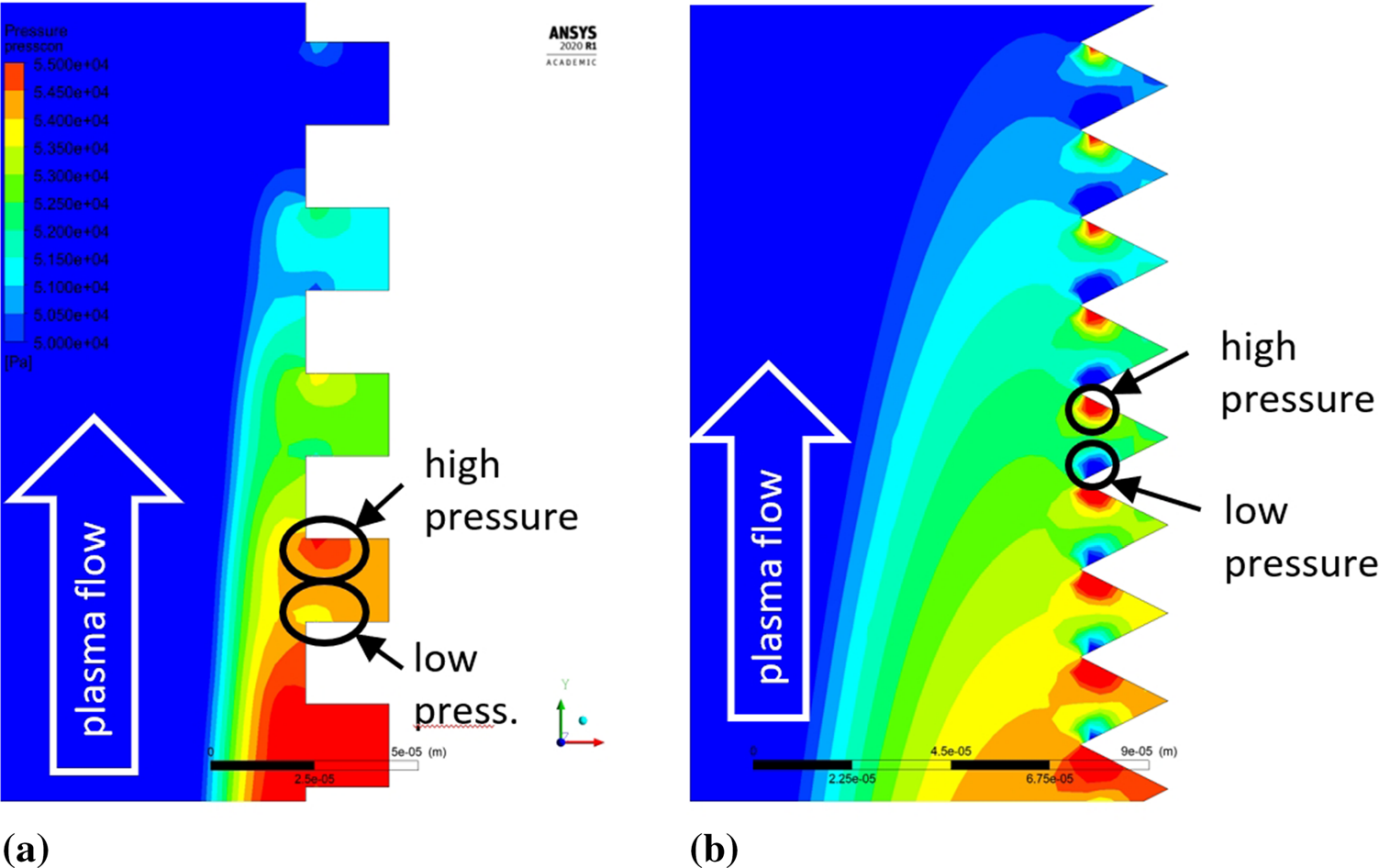
Local pressure field distribution along (a) rectangular substrate surface topography, (b) V-shape substrate topography; (the axial and radial scale is equal)

**Fig. 18:**
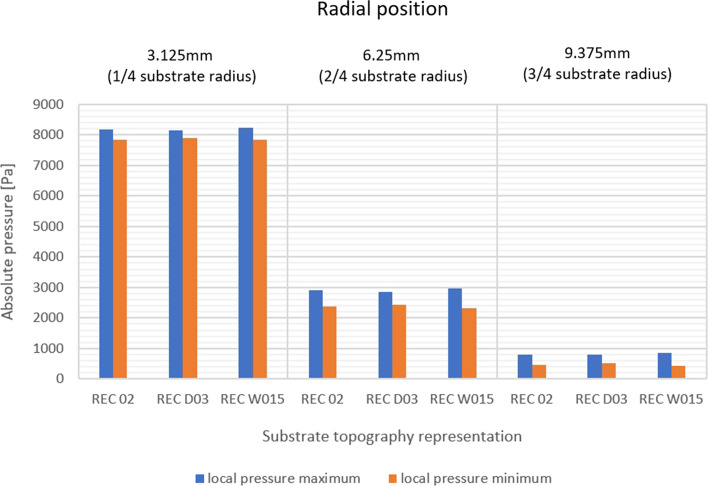
Local in-groove pressure variation for rectangular substrate representations

**Fig. 19:**
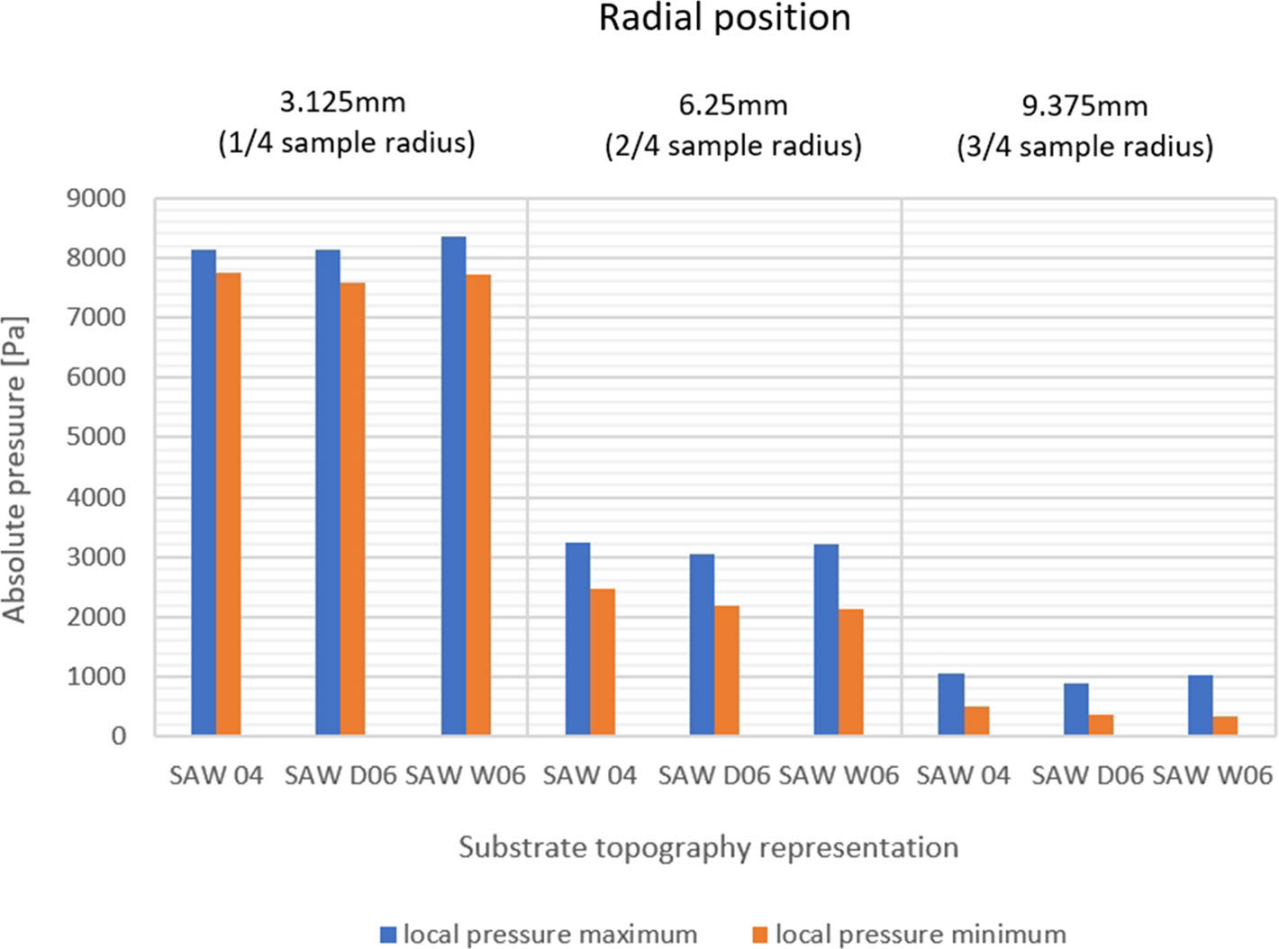
Local in-groove pressure variation for V-shape substrate representations

**Fig. 20:**
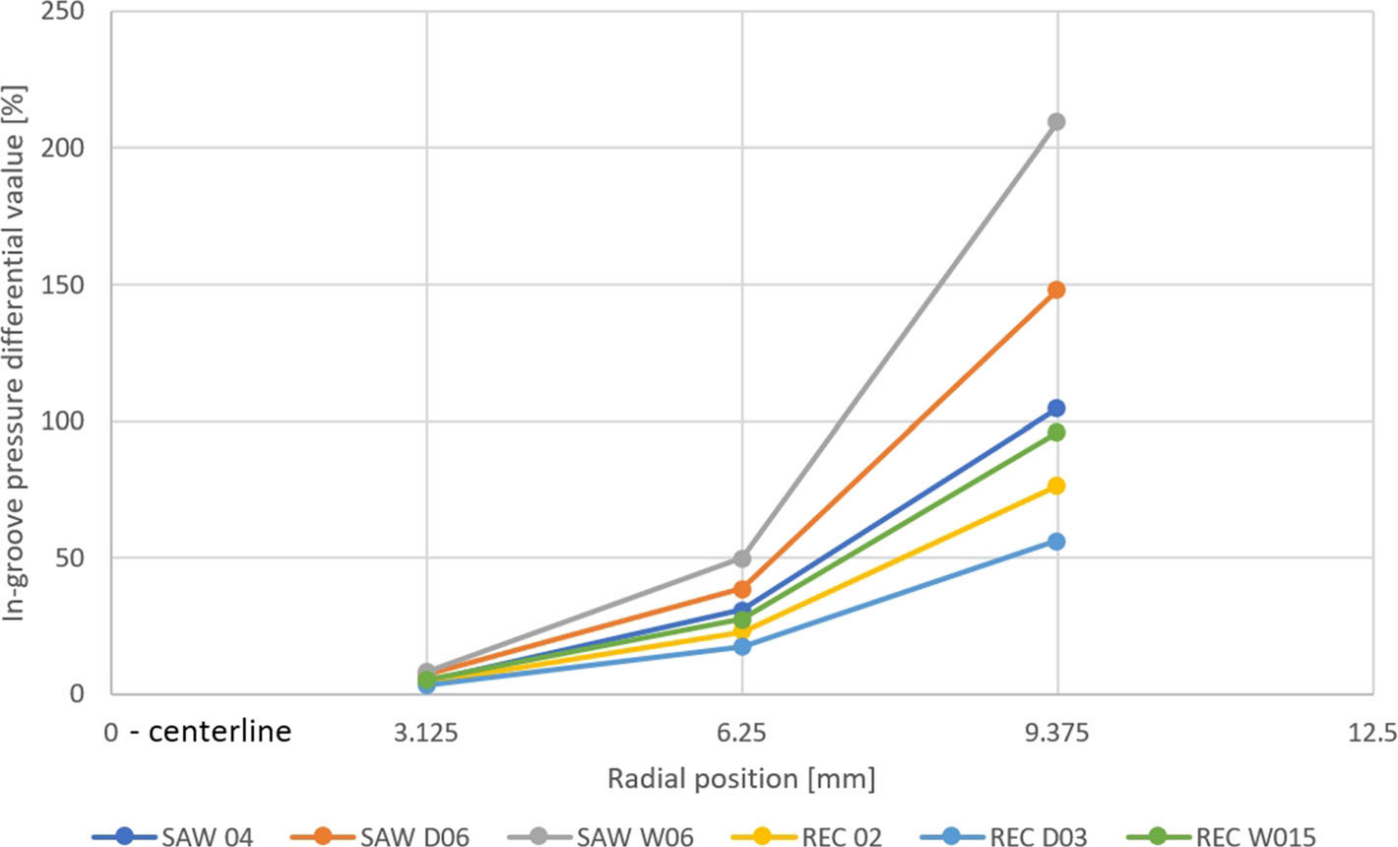
Local in-groove pressure differential percentage value

The pressure field analysis corresponds with the previous observations of the behavior of the plasma jet in the surface boundary region. The stream hitting the vertical groove wall causes a local increase in pressure from the leading side and a slight pressure drop from the trailing side (Fig. 17). Three types of rectangular and three types of V-shape substrate profiles were investigated here to determine the influence of small variations in the substrate topography geometrical parameters on the plasma stream behavior, and thus, the feedstock deposition mechanisms (Table 2). The analysis of the pressure field in the substrate boundary layers showed that the pressure value depends primarily on the radial distance from the substrate centerline. Although the overall pressure values measured at certain measure points (Fig. 18 and 19) seem to be independent of the substrate topography, the mentioned local pressure field distribution differs between the topography representations. Figure 20 shows the pressure differential percentage value $\Delta P_{\% }$ between the local maximum and minimum pressure in terms of radial position of the measuring point for all topography variants. $\Delta P_{\% }$ is calculated from:

10 $$\Delta P_{\% } = \frac{{P_{h} - P_{l} }}{{P_{l} }} \cdot 100\% ,$$

where $P_{h}$ is the maximum and $P_{l}$ is the minimum value of the local pressure inside the groove. It was determined that the substrate with a V-shape topography generated a greater $\Delta P_{\% }$ than the rectangular one, but for both types of substrates, greater groove width resulted in a slightly higher $\Delta P_{\% }$. Additionally, the analysis of the differential percentage value showed that the groove width effect is more significant for low pressure regions located far from the substrate axis and the global stagnation area (Fig. 20). The depth of the groove seems to not influence the pressure field distribution in the substrate boundary layer significantly.

**Fig. 21:**
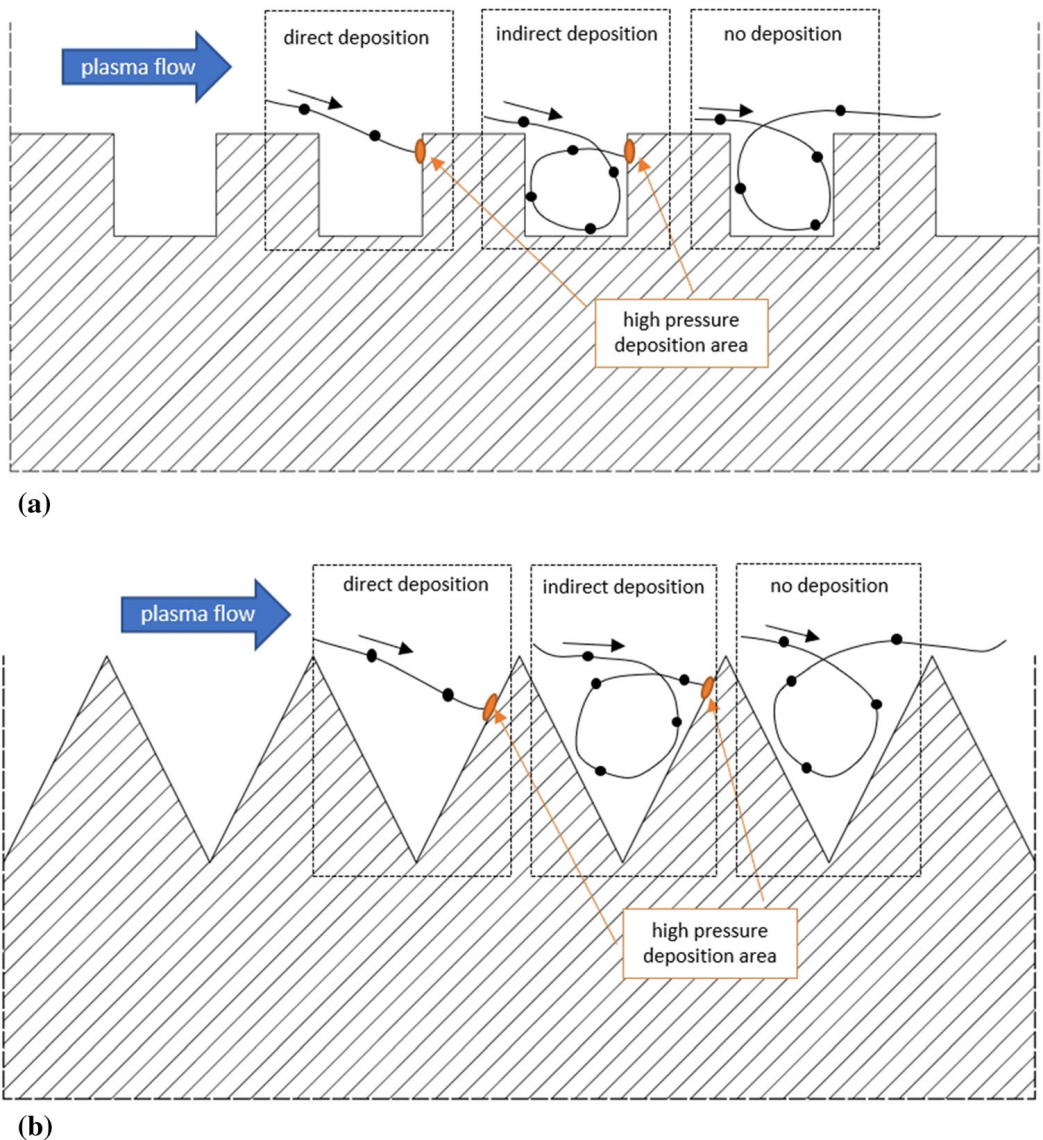
Scheme of predicted particle trajectories and deposition mechanisms in contact with (a) rectangular substrate topography and (b) V-shape substrate topography

In general, the results of a numerical simulation may be helpful to discuss the build-up mechanism in plasma spraying. In this particular work, the results are discussed in the context of SPS sprayed TBC over microtexturized bond coats because such coatings will be developed in the further stages of our work. Kromer et al. (Ref 6) confirmed experimentally that micro-scale texturization of substrates allows the changing of Suspension Plasma Sprayed TBC structure. The analyses discussed in this paper confirm that there is an important difference in the plasma jet versus substrate interaction while depositing coatings on flat or textured surfaces. The distribution of the plasma kinetic energy field in the sample’s near-wall area affects directly the energy of the fine, sub-micrometer, particles moving in the plasma jet due to their low Stokes number (Ref 2, 30). The maximum kinetic energy in the boundary layer of the flat substrate is lower for a flat substrate when compared to a microtextured one (Fig. 15). As mentioned above, the plasma jet’s swirl inside the grooves resulted in a much lower velocity than for the stream flowing over the top of the grooves. For this reason, the plasma volume swirling inside the groove cannot return into the main stream. This phenomenon and the fact that there is a high-pressure area located near the groove edge on the leading side is supposed to intensify the TBC columnar structure build-up mechanism, as intended. Consequently, the first layer of the coating is deposited on the groove’s leading edge rather than on the leeward edge or on the bottom of the groove. This was already confirmed experimentally by Caio et al. (Ref 31), Kromer et al. (Ref 6), and Sokołowski et al. (Ref 32), who worked on the deposition of the columnar structure by the SPS process. In those studies, the column formation mechanism was mainly controlled by ensuring a proper substrate topography. Figure 21 shows the predicted molten particle trajectories and three deposition possibilities for both modeled substrate topography representations. If the deposition occurs, the particle can be deposited either directly without following the vortex plasma flow inside the groove or indirectly following plasma swirl flow as presented in Fig. 21.

Moreover, the laser microtexturization prior to the spraying opens the possibility of precise controlling the substrate topography, which may be used for tailoring the columnar-like TBCs. Although the suspended feedstock contains the majority of sub-micrometer particles, they vary in size and, consequently, in their Stokes number. It is known that the small particles with a subcritical Stokes number (St<1) are more likely to impact the side of the substrate or to be carried away while bigger particles deposit closer to the centerline and the stagnation area (Ref 2). VanEvery et al. (Ref 3) investigated this correlation between drag forces and the YSZ particle size in the substrate boundary layer. The velocity normal to the substrate varied between 400 m/s for 40 µm to 50 m/s for 1 µm particles measured at 0.1 cm above the substrate. Another experimental approach in such a field is the analysis of the pressure exerted on the substrate by impinging particles proposed by Dolmaire et al. (Ref 29). The splat-forming pressure decreases with the particle incidence angle, which corresponds to the radial position of the impinging particle. It proves that the relatively high Stokes number particles are being deposited near the centerline rather than in the outer regions of the substrate resulting in a higher impinging pressure and a higher density of the deposited coating. The analysis of particle deceleration resulting from the substrate presence conducted by Dolmaire et al. (Ref 33) is worth to mention here as well. It is shown that, due to low sub-micrometers YSZ particles Stokes numbers between 0.35 and 0.10, the sensitivity to the plasma flow is increased. The mentioned analyses support the idea of microtexturization as an effective tool to control the low-Stokes particles deposition and movement in the substrate boundary layer.

The results discussed in this section demonstrated that the variation of the substrate profile geometry is considerable, especially in the outer regions of the substrate. Far from the substrate centerline, the pressure distribution depends significantly on the microtextured groove width. Increasing this parameter by 50%, from 40 to 60 µm, doubled the local pressure differential value between the leading and trailing side of the groove for the V-shape topography representation. In those side areas of the substrate, the local pressure field variations significantly affect the trajectories of particles as their Stokes number is relatively low. Hence, the smaller particles are being deposited far from the substrate centerline. This showed also the usefulness of the presented numerical simulation studies, which may help in the preliminary selection of proper substrate topography for getting the desired coating morphology and, in turn, limit the experimental work necessary for coating development.

## Conclusions

In this work, the influence of the substrate topography on the plasma behavior in the substrate boundary layer was numerically investigated. The numerical model was developed on the idea of using the laser ablation for MCrAlY microtexturization and the deposition of fine, and controlled, columnar-like TBCs by Suspension Plasma Spraying.

The results showed that the substrate topography influences the plasma jet flow in the substrate boundary layer. The effect is mainly noticeable in the side areas of the substrate, where the relative local pressure fluctuations are significant. The local pressure differential percentage values for the different topographies were analyzed. The V-shape topography representation, e.g. SAW_06, reaches 200% in 9.375 mm from the substrate centerline, while at the 3.125 mm measuring point, there is an 8% pressure differential value. This trend is also observed for rectangular representations, e.g. REC_W15. In this case, at the 3.125 mm measuring point, the local pressure differential percentage value is 5%, while at 9.375 mm point, it reaches 96%. This, in turn, proves that the sample topography may be predominant in terms of coating build-up mechanisms in plasma spraying. Fine powders in SPS are especially sensitive to the discussed phenomena, as these particles deposited in low-pressure regions are characterized by a low Stokes number. This effect is also intensified by the local swirl of plasma taking place in each fine texture (V-shape or rectangular one) created by laser ablation. This affects the morphology of plasma sprayed coating and may be used to better control it.

However, further analyses are needed for a better understanding of the TBC build-up mechanism. The future studies will include topographies with a greater groove width variation to extrapolate the effect to higher Stokes number particles. Then, the feedstock injection as well as injection related phenomena, e.g.: solvent evaporation, particles melting, and aggregating as well as particle drag influence on the plasma flow will be considered for further developing the numerical model. As discussed in this paper, the numerical analyses may be used to reduce the number of initial experimental iterations required to obtain a high performance columnar TBC, which is planned for the future as well.
